# Determinants of cancer screening awareness and participation among Indonesian women

**DOI:** 10.1186/s12885-018-4125-z

**Published:** 2018-03-06

**Authors:** Sumadi L. Anwar, Gindo Tampubolon, Mieke Van Hemelrijck, Susanna H. Hutajulu, Johnathan Watkins, Wahyu Wulaningsih, for the PILAR Research Network

**Affiliations:** 1 Division of Surgical Oncology Department of Surgery, Faculty of Medicine Universitas Gadjah Mada, Yogyakarta, 55281 Indonesia; 2 PILAR Research and Education, 20 Station Road, Cambridge, CB1 2JD UK; 3 Cathie Marsh Institute for Social Research, University of Manchester, M13 9PL, Manchester, UK; 4 Translational Oncology and Urology Research, King’s College London, London, SE1 9RT UK; 5 Division of Medical Haematology and Oncology Department of Internal Medicine, Faculty of Medicine Universitas Gadjah Mada, Yogyakarta, 55281 Indonesia; 6 MRC Unit for Lifelong Health and Ageing, University College London, Place London WC1B 5JU, Bedford, 33 UK

**Keywords:** Breast cancer, Screening, Cervical cancer, Pap smears, Breast self-examination

## Abstract

**Background:**

Cancer screening awareness and participation may be lower in low- and middle-income countries that lack established national screening programmes compared with those that do. We evaluated potential determinants of awareness about and participation in breast and cervical cancer screening, and breast self-examination (BSE) in women using survey data from Indonesia.

**Methods:**

From the fifth Indonesian Family Life Survey (2014–2015), a total of 5397 women aged 40 and older without any history of cancer who responded to questionnaires concerning Pap smears, mammography, and BSE were included. Multilevel modelling was used to assess potential determinants in relation to awareness about Pap smears and mammography, and participation in Pap smears and BSE practice. Multivariable analyses were performed to identify independent predictors of cancer screening.

**Results:**

Of the 5397 respondents, 1058 (20%) women were aware of Pap smears, of which 297 had never had the procedure. Only 251 (5%) participants were aware of mammography. A total of 605 (12%) of women reported they performed BSE. Higher education and household expenditure were consistently associated with higher odds of awareness about Pap smears and mammography (e.g. odds ratio [OR] of being aware of Pap smear and mammography: 7.82 (95% CI: 6.30–9.70) and 7.70 (6.19–9.58), respectively, for high school graduates compared to women with less educational attainment in the multivariable models), and participation in Pap smears and BSE. We also identified enabling factors linked with greater cancer screening awareness and participation, including health insurance, shorter distance to health services, and social participation.

**Conclusion:**

There are socioeconomic disparities in cancer screening awareness and participation among Indonesian women. Our findings may help inform targeted health promotion and screening for cancer in the presence of limited resources.

**Electronic supplementary material:**

The online version of this article (10.1186/s12885-018-4125-z) contains supplementary material, which is available to authorized users.

## Background

The overall burden of cancer has been increasing in developing countries [1]. The World Health Organization (WHO) International Agency for Research on Cancer (IARC) estimated that there will be up to 21.7 million new cancer cases and 13 million cancer-related deaths in 2030, with 70% of those cases in low- to middle-income countries (LMICs) [2–4]. Although cancer mortality rates have declined in high-income countries, LMICs have seen elevated cancer-related mortality rates [5], owing to a lack of cancer prevention and screening programmes and limited resources to treat cancer [4, 6].

In LMICs such as Indonesia, cancers are mostly diagnosed at an advanced stage, in which curative treatment is often no longer possible [7]. For female cancers, breast and cervical cancers remain the leading causes of cancer mortality in Indonesia (21% and 10%, respectively) [5]. Yet, affordable cervical cancer screening is only available in eight of 34 provinces in Indonesia, [5, 8] with low awareness and uptake of breast and cervical cancer screening [5, 8, 9]. The low uptake may be attributable to a range of barriers including a lack of knowledge about cancer prevention as well as widespread misconceptions and fears about cancer and its treatment [9, 10] also further contribute to the late presentation of disease [11]. In addition, there are often inequalities in the distribution of healthcare workers throughout the country, resulting in inequalities in healthcare access especially between urban and rural areas [12]. Nonetheless, the extent of inequalities in cancer screening awareness and participation in LMICs, such as Indonesia, is often unclear. Additionally, breast self-examination (BSE) as a tool to screen for breast cancer is common in these countries, although there is evidence to suggest that this technique lacks effectiveness [13].

We performed a cross-sectional study of 5397 cancer-free Indonesian women aged 40 and older, the target group for breast and cervical cancer screening based on American Cancer Society Guidelines [14]. We used multilevel regression analyses to identify potential determinants of cervical and breast cancer screening awareness and participation to gain further insight into predisposing, enabling, and need factors which could potentially inform targeted prevention programmes in low-resource settings.

## Methods

### Study population

The Indonesian Family Life Survey (IFLS) is a longitudinal household survey in Indonesia containing information from questionnaires, as well as physical and laboratory examinations. Data were collected at individual, household, and community levels. The first IFLS (IFLS1) used a stratified sampling scheme based on provinces and urban/rural location. For cost-effectiveness, 14 of the 27 provinces that existed at the time IFLS1 was conducted were excluded [15]. The resulting sample included 13 of Indonesia’s 27 provinces, containing 83% of the population: four provinces on Sumatra (North Sumatra, West Sumatra, South Sumatra, and Lampung), all five of the Javanese provinces (DKI Jakarta, West Java, Central Java, DI Yogyakarta, and East Java), and four provinces covering the remaining major island groups (Bali, West Nusa Tenggara, South Kalimantan, and South Sulawesi). Within each province, enumeration areas (EAs) were randomly chosen from a nationally representative sample frame used in a socioeconomic survey of about 60,000 households in 1993 [15]. Within a selected EA, households were randomly selected. Interviews were carried out with the household head and the spouse, and up to 4 randomly selected other household members as interviewing all members of the household would have been too costly. All members of the original household were followed up through four subsequent IFLS waves. The present study was based on the fifth wave of IFLS (IFLS5), conducted in 2014–2015. Both original and split-off households were tracked in the IFLS5, resulting in a 76% re-contact rate (including death) for the original IFLS1 household members, and 82% for IFLS1 main respondents. From the IFLS5, we included a total of 5397 women aged 40 and older without a self-reported history of cancer who responded to the questions on Pap smears, mammography, and BSE (Additional file 1: Fig. S1).

### Cancer screening participation and behaviour

The outcomes of the present study measured awareness of Pap smears and mammography, and participation in Pap smears and BSE practice. All responses were self-reported and dichotomous (yes, no). First, during the interview, respondents were asked if they had ever heard of a Pap smear. Those who responded positively to this question were further asked whether they had ever received a Pap smear in their life, and, if so, when. Participants were also asked whether they had ever heard about mammography, and those who responded positively were asked whether they had ever received a mammography in the past year. All study participants were asked how many times they had performed BSE in the past year, and we further dichotomised them into those who had performed BSE and who had not.

### Potential determinants of screening

**Fig. 1:**
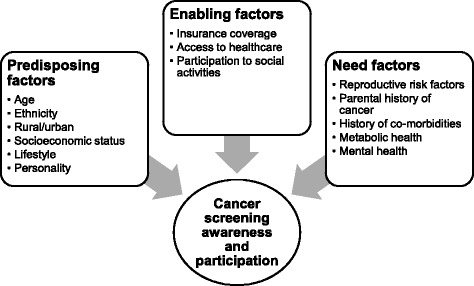
Potential determinants of cancer screening awareness and participation in IFLS5 based on the Anderson model of health behaviour

The Anderson model of healthcare-use behaviour [16] was used to identify potential determinants of cancer screening awareness and participation. This model includes three domains: predisposing, enabling, and need factors, which interact in determining one’s health-related behaviour. From IFLS5 (Fig. 1), predisposing sociodemographic and lifestyle characteristics of the patients were collected including age, ethnicity, urban or rural residence, marital status, education, monthly household expenditure, smoking status, physical activity and personality traits. Ethnicity was categorised into Javanese, which comprises the majority of Indonesians, and non-Javanese. Education was categorised based on the highest level (less than high school, high school, higher education). Household expenditure was calculated based on the total of food, non-food, and education expenditure [17]. Smoking history was used to classify individuals into current, former, and never smokers. Participants were defined as vigorously active if they reported participating in more than two vigorous physical activities in the past week for at least 10 min each, [18] moderately active if they participated more than 4 times in the past week in moderate to vigorous physical activities of which no more than two could be considered vigorous. Lightly active was defined as participating in any activities or walking at least 30 min each time, for more than 2 times in the past week but did not meet the description of vigorously or moderately active. Those who reported no moderate or vigorous physical activity and walked fewer than 3 times a week were categorised as sedentary. Personality traits were assessed with a short (15-item) Big Five Inventory (BFI-15) questionnaire [19], with scores ranging from 1 to 5 for openness, conscientiousness, extroversion, agreeableness, and neuroticism.

Enabling factors identified in the population included health insurance ownership and travel distance in minutes to the nearest healthcare centre, and participation in any social activities within the past year. On 1 January 2014, the Indonesian government launched a compulsory national health insurance,which covers Pap smears [20], although this scheme has yet to cover mammography. However, unequal healthcare access issues were reported within the first year of the scheme being implemented (2014–2015) [21], which is the period in which the present study took place. Therefore, we took into account self-reported insurance coverage in our analysis. Factors representing needs for cancer screening included information on reproductive factors: menopausal status, age at menarche, co-morbidities, parental history of cancer death, and body mass index (BMI) calculated from measured weight and height during physical examination. Co-morbidities were assessed as a comorbidity score similar to the Charlson co-morbidity index, where each co-morbid condition available (hypertension, diabetes, asthma, heart disease, liver disease, stroke, cancer, arthritis, kidney disease, stomach or digestive disease, and memory-related disease) contributed one point to the composite index with additional points given for older age. Finally, to assess the role of mental health, depression was measured with a short version (10-item) of the Center for Epidemiologic Studies Depression Scale (CES-D), [15] and a cut-off of 8 was used for a screening of depressive symptoms [19].

### Statistical analysis

We analysed the association between each determinant with awareness of Pap smears, awareness of mammography, ever-Pap smear, and performed BSE. We did not assess use of mammography as an outcome given the small number of participants with a positive response. To take into account the IFLS sampling design, we performed multilevel logistic regression analyses to obtain odds ratios (ORs) and their 95% CI for associations between each determinant and outcome. Community clustering was used as a random effect in a two-level multivariable model. Household clustering was not used in the multilevel model due to there being an inadequate number of participants for generating meaningful statistical results for a number of factors. However, where possible, we compared models using both community and household clustering with models using only community clustering with analysis of co-variance, and no difference was observed between these models (*P* > 0.05). Univariable analyses were conducted for all potential determinants of cancer screening awareness and practice. These factors comprised different components of the Anderson model [16], ranging from predisposing factors such as sociodemographic characteristics (e.g. marital status, education, income), enabling factors, which included healthcare access. We additionally included comorbid conditions (e.g. diabetes, obesity as measured by BMI) given the evidence linking comorbidity to cervical and breast cancer screening participation in Western populations [22]. Multivariable analyses were further carried out and included factors which showed significant associations with cancer awareness or participation in the univariable models. We performed a sensitivity analysis by grouping participants into those who had performed BSE more than once and those who had. The dataset was prepared with SAS release 9.3 (SAS Institute, Cary, NC). Logistic regression with multilevel modelling was performed with the *lme4* package in R version 3.3.2 (R Foundation for Statistical Computing, Vienna, Austria).

## Results

Characteristics of the study participants (*N* = 5397) are presented in Table 1. The mean age of participants was 52.9 years. The majority of women were of Javanese ethnicity, married, lived in urban areas, and had not completed high school. Nearly a quarter (23%) of women had three or more co-morbidities, and a similar proportion were overweight (BMI ≥ 25 kg/m^2^). Only 1058 (20%) women were aware of Pap smears and 297 among them had undergone at least one Pap smear in their lifetime. A total of 251 (5%) participants were aware of mammography, among which five had had a mammogram in the previous year. Twelve percent of women reported they had performed BSE in the past year. We additionally present the demographic characteristics of women who did not respond to questions on cancer screening (Additional file 1: Table S1), which comprised 9.6% of women aged 40 and older. Compared to women who provided a response to cancer screening, non-responders were in average older, less educated, had lower household expenditure, and more likely to be non-Javanese or unmarried.

**Table 1:** Characteristics of study participants (*N* = 5397)

Potential determinants	N (%)	
Predisposing		
Age	40–60	4098 (75.93)
	≥60	1299 (24.07)
Ethnicity	Not Javanese	2900 (43.73)
	Javanese	2497 (46.27)
Residence	Rural	2339 (43.34)
	Urban	3058 (56.66)
Marital status	Not married	1423 (26.37)
	Married	3974 (73.63)
Education	Less than high school	3534 (65.48)
	High school	1529 (28.33)
	Higher education	334 (6.19)
Monthly household expenditure	Tertile 1–2	3561 (65.98)
	Tertile 3	1836 (34.02)
Tobacco smoking	Never	5157 (95.55)
	Former	63 (1.17)
	Ever	177 (3.28)
Physical activity	Sedentary	1627 (30.15)
	Lightly active	1793 (33.22)
	Moderately active	1460 (27.05)
	Vigorously active	517 (9.58)
Openness	< 4	4715 (87.36)
	≥4	682 (12.64)
Conscientiousness	< 4	4690 (86.90)
	≥4	707 (13.10)
Extroversion	< 4	3203 (59.35)
	≥4	2194 (40.65)
Agreeableness	< 4	1771 (32.81)
	≥4	3626 (67.19)
Neuroticism	< 4	3191 (59.13)
	≥4	2206 (40.87)
Enabling		
Insured	No	2754 (51.03)
	Yes	2643 (48.97)
Travel time	< 10 min	4503 (83.44)
	≥10 min	894 (16.56)
Participating in social activities	No	806 (14.93)
	Yes	4591 (85.07)
Need		
Menopausal status	Premenopausal	2300 (42.61)
	Postmenopausal	3097 (57.38)
Age at menarche	< 14	2133 (39.52)
	≥ 14	3264 (60.48)
Co-morbidity score	0	1476 (27.35)
	1	1506 (27.90)
	2	1162 (21.53)
	3 and more	1253 (23.22)
Parent died from cancer	No	5264 (97.54)
	Yes	133 (2.46)
BMI	< 25 kg/m^2^	2879 (53.34)
	≥ 25 kg/m^2^	2518 (46.66)
CES-D	< 8	5097 (94.44)
	≥ 8	300 (5.66)

### Determinants of awareness of pap smears

Table 2 shows potential determinants of awareness of Pap smears identified through univariable regressions and grouped according to the Anderson model. Some categories, for instance education levels, were merged in the analysis due to the limited numbers of participants. In the analysis, age, ethnicity, urban residence, marital status, education level, household expenditure, physical activity, openness, extroversion, agreeableness, neuroticism, insurance, distance to healthcare providers, menopausal status, age at menarche, comorbidity score, parental deaths of cancer, overweight, and CESD were associated with awareness of Pap smears. In the multivariable analysis (Table 4), being Javanese (OR: 1.91, 95% CI: 1.52–2.40), living in urban area (OR: 4.28, 3.22–5.67), graduating high school (OR: 7.82, 6.30–9.70), greater household expenditure (OR: 2.31, 1.91–2.80), physical activity (OR: 1.54, 1.25–1.91), agreeable (1.63, 1.30–2.03) and neuroticism traits (OR: 1.23, 1.02–1.49), having insurance (OR: 2.05, 1.69–2.49), and participating in social activities (OR: 2.12, 1.50–2.98) corresponded to higher likelihood of being aware of Pap smears. As shown in Table 4, a decrease in the odds of Pap smear awareness was shown as the distance to a healthcare provider increased (OR: 0.73, 0.55–0.98) and CESD score (OR: 0.68, 0.55–0.85) in the multivariable model.

**Table 2:** Univariable associations of potential determinants with cancer screening awareness among women 40 years and older without known history of any cancer

Potential determinants		Aware of Pap smear (N = 5397)		Aware of mammography (N = 5397)	
		N (%)	OR (95% CI)	N (%)	OR (95% CI)
Predisposing					
Age	40–60	919 (22.42)	Ref	220 (5.37)	Ref
	≥60	139 (10.70)	0.32 (0.25–0.40)	31 (2.39)	0.44 (0.43–0.45)
Ethnicity	Not Javanese	478 (16.48)	Ref	146 (5.03)	Ref
	Javanese	580 (23.23)	1.49 (1.18–1.88)	105 (4.21)	0.85 (0.59–1.22)
Residence	Rural	166 (7.09)	Ref	54 (2.31)	Ref
	Urban	892 (29.16)	7.74 (5.77–10.37)	197 (6.44)	3.57 (2.34–5.44)
Marital status	Not married	193 (13.56)	Ref	43 (3.02)	Ref
	Married	865 (21.77)	2.14 (1.74–2.62)	208 (5.23)	1.93 (1.34–2.75)
Education	Less than high school	223 (5.31)	Ref	70 (1.98)	Ref
	High school or higher education	835 (44.82)	14.01 (11.44–17.16)	181 (9.72)	5.08 (3.75–6.90)
Monthly household expenditure	Tertile 1–2	446 (12.52)	Ref	95 (2.67)	Ref
	Tertile 3	612 (33.33)	3.66 (3.08–4.35)	156 (8.50)	3.29 (2.49–4.33)
Tobacco smoking	Never	1019 (19.76)	Ref	241 (4.67)	Ref
	Ever	39 (16.25)	0.78 (0.50–1.21)	10 (4.17)	0.87 (0.43–1.76)
Physical activity	Active	268 (16.47)	Ref	51 (3.13)	Ref
	Sedentary	790 (20.95)	1.49 (1.23–1.80)	200 (5.31)	1.85 (1.32–2.60)
Openness	< 4	957 (20.30)	Ref	226 (4.79)	Ref
	≥4	101 (14.81)	0.77 (0.59–1.00)	25 (3.67)	0.94 (0.62–1.44)
Conscientiousness	< 4	929 (19.81)	Ref	218 (4.64)	Ref
	≥4	129 (18.25)	1.00 (0.78–1.28)	33 (4.67)	1.02 (0.69–1.53)
Extroversion	< 4	688 (21.48)	Ref	167 (5.21)	Ref
	≥4	370 (16.86)	0.75 (0.63–0.88)	84 (3.83)	0.76 (0.57–1.00)
Agreeableness	< 4	199 (11.24)	Ref	50 (2.82)	Ref
	≥4	859 (23.69)	2.53 (2.08–3.08)	201 (5.54)	1.91 (1.37–2.64)
Neuroticism	< 4	528 (16.55)	Ref	111 (3.48)	Ref
	≥4	530 (24.03)	1.65 (1.40–1.95)	140 (6.35)	1.88 (1.87–1.89)
Enabling					
Insured	No	340 (12.35)	Ref	82 (2.98)	Ref
	Yes	718 (27.17)	2.52 (2.11–3.01)	169 (6.39)	2.10 (1.57–2.82)
Travel time	< 10 min	952 (21.14)	Ref	232 (5.15)	Ref
	≥10 min	106 (11.86)	0.48 (0.37–0.62)	19 (2.12)	0.42 (0.26–0.70)
Participating in social activities	No	65 (8.06)	Ref	20 (2.48)	Ref
	Yes	993 (21.63)	3.27 (2.39–4.47)	231 (5.03)	2.25 (1.35–3.75)
Need					
Menopausal status	Premenopausal	641 (27.87)	Ref	160 (6.96)	Ref
	Postmenopausal	417 (13.46)	0.36 (0.30–0.42)	91 (2.94)	0.41 (0.31–0.54)
Age at menarche	< 14	472 (22.13)	Ref	115 (5.39)	Ref
	≥ 14	586 (17.95)	0.77 (0.65–0.91)	136 (4.17)	0.73 (0.56–0.95)
Co-morbidity score	0–1	692 (23.21)	Ref	179 (6.00)	Ref
	≥ 2	366 (15.15)	0.53 (0.45–0.63)	72 (2.98)	0.54 (0.40–0.72)
Parent died from cancer	No	1009 (19.17)	Ref	238 (4.52)	Ref
	Yes	49 (36.84)	2.58 (1.62–4.11)	13 (9.77)	2.25 (1.15–4.41)
BMI	< 25 kg/m^2^	457 (15.87)	Ref	121 (4.20)	Ref
	≥ 25 kg/m^2^	601 (23.87)	1.49 (1.26–1.76)	130 (5.12)	1.12 (0.85–1.48)
CES-D	< 8	820 (21.42)	Ref	190 (4.96)	Ref
	≥ 8	238 (15.18)	0.73 (0.60–0.88)	61 (3.89)	0.86 (0.63–1.17)

### Determinants of awareness of mammography

Similar patterns of associations between potential predictors and awareness of Pap smears were observed for awareness of mammography in the univariable analysis (Table 2). In the multivariable model, we found higher odds of being aware of mammography in women living in urban areas (OR: 4.51, 95% CI: 3.36–6.06), women who had graduated high school (OR: 7.70, 6.19–9.58), women with higher household expenditure (OR: 2.28, 1.88–2.76), women that do physical activity (OR: 1.54, 1.24–1.90), women who have greater agreeableness (OR: 1.67, 1.33–2.09), women with neuroticism traits (OR: 1.24, 1.03–2.09), women who have insurance (OR: 2.01, 1.65–2.44), and women who participate in social activities (OR: 2.29, 1.62–3.23) (Table 4). Living further from health services (OR: 0.70, 0.52–0.94) and being postmenopausal (OR: 0.79, 0.63–0.99) were inversely associated with being aware of mammography in the multivariable model.

### Determinants of pap smear participation

We assessed factors associated with participation in Pap smears (Table 3), and only found education level, household expenditure, insurance, menopausal status and comorbidity score to be associated with participation in Pap smears in the univariable analysis. In the multivariable models, women were more likely to have had a Pap smear if they had graduated high school (OR: 1.58, 95% CI: 1.04–2.41), had higher household expenditure (OR: 1.94, 1.40–2.69), had insurance (1.57 (1.12–2.22), and had two or more co-morbidities (1.45, 1.01–2.08) (Table 4).

**Table 3:** Univariable associations of potential determinants with cancer screening practice among women 40 years and older without known history of any cancer

Potential determinants		Ever Pap smear (*N* = 1058)		Performed BSE (N = 5397)	
		N (%)	OR (95 CI)	N (%)	OR (95 CI)
Predisposing					
Age	40–60	254 (27.64)	Ref	538 (13.13)	Ref
	≥60	43 (30.94)	1.06 (0.55–2.03)	67 (5.16)	0.33 (0.25–0.43)
Ethnicity	Not Javanese	133 (27.82)	Ref	302 (10.41)	Ref
	Javanese	164 (28.28)	0.58 (0.72–1.33)	303 (12.13)	1.20 (0.96–1.51)
Residence	Rural	50 (30.12)	Ref	129 (5.52)	Ref
	Urban	247 (27.69)	0.85 (0.56–1.28)	476 (15.56)	3.54 (2.74–4.58)
Marital status	Not married	52 (26.94)	Ref	99 (6.96)	Ref
	Married	245 (28.32)	1.10 (0.76–1.60)	506 (12.73)	2.06 (1.62–2.61)
Education	Less than high school	48 (21.52)	Ref	141 (3.99)	Ref
	High school or higher education	249 (29.82)	1.70 (1.16–2.50)	464 (24.91)	8.24 (6.67–10.18)
Monthly household expenditure	Tertile 1–2	92 (20.63)	Ref	265 (7.44)	1.70 (1.54–1.88)
	Tertile 3	205 (33.50)	2.08 (2.07–2.09)	340 (18.52)	
Tobacco smoking	Never	286 (28.07)	Ref	580 (11.25)	Ref
	Ever	11 (28.21)	1.06 (0.50–2.26)	25 (10.42)	0.95 (0.60–1.51)
Physical activity	Active	72 (26.87)	Ref	157 (9.64)	Ref
	Sedentary	225 (28.48)	1.12 (0.80–1.56)	448 (11.88)	1.30 (1.06–1.60)
Openness	< 4	272 (28.42)	Ref	544 (11.54)	Ref
	≥4	25 (24.75)	0.89 (0.55–1.45)	61 (8.94)	0.79 (0.59–1.07)
Conscientiousness	< 4	264 (28.42)	Ref	531 (11.32)	Ref
	≥4	33 (25.58)	0.83 (0.53–1.29)	74 (10.47)	0.95 (0.72–1.25)
Extroversion	< 4	199 (28.92)	Ref	400 (12.49)	Ref
	≥4	98 (26.49)	0.96 (0.72–1.30)	205 (9.34)	0.74 (0.61–0.90)
Agreeableness	< 4	59 (29.65)	Ref	107 (6.04)	Ref
	≥4	238 (27.71)	0.97 (0.69–1.39)	498 (13.73)	2.48 (1.98–3.11)
Neuroticism	< 4	135 (25.57)	Ref	294 (9.21)	Ref
	≥4	162 (20.57)	1.31 (0.99–1.74)	311 (14.09	1.61 (1.34–1.93)
Enabling					
Insured	No	76 (22.35)	Ref	217 (7.87)	Ref
	Yes	221 (30.78)	1.70 (1.23–2.37)	388 (14.68)	1.97 (1.63–2.39)
Travel time	< 10 min	275 (28.87)	Ref	545 (12.10)	Ref
	≥10 min	22 (20.75)	0.61 (0.36–1.02)	60 (6.71)	0.52 (0.39–0.70)
Participating in social activities	No	13 (20.00)	Ref	38 (4.71)	Ref
	Yes	284 (28.60)	1.56 (0.81–3.00)	567 (12.35)	2.85 (1.99–4.08)
Need					
Menopausal status	Premenopausal	163 (25.42)	Ref	403 (17.52)	Ref
	Postmenopausal	134 (32.13)	1.38 (1.04–1.85)	202 (6.52)	0.32 (0.26–0.38)
Age at menarche	< 14	138 (29.24)	Ref	274 (12.85)	Ref
	≥ 14	159 (27.13)	0.87 (0.65–1.15)	331 (10.14)	0.80 (0.66–0.96)
Co-morbidity score	0–1	73 (25.29)	Ref	412 (13.82)	Ref
	≥ 2	58 (33.33)	1.58 (1.18–2.12)	193 (7.99)	0.52 (0.43–0.63)
Parent died from cancer	No	281 (27.85)	Ref	573 (10.89)	Ref
	Yes	16 (32.65)	1.30 (0.68–2.50)	32 (24.06)	2.42 (1.54–3.83)
BMI	< 25 kg/m^2^	119 (26.04)	Ref	264 (9.17)	Ref
	≥ 25 kg/m^2^	178 (29.62)	1.18 (0.89–1.58)	341 (13.54)	1.44 (1.20–1.74)
CES-D	< 8	235 (28.66)	Ref	439 (11.47)	Ref
	≥ 8	62 (26.05)	0.91 (0.65–1.28)	166 (10.59)	0.99 (0.81–1.22)

**Table 4:** Multivariable associations of potential determinants with cancer screening awareness among women 40 years and older without known history of any cancer

Potential determinants^a^	OR (95% CI)			
	Aware of pap smear	Aware of mammography	Ever pap smear	Performed BSE
Predisposing				
Age	0.72 (0.52–0.99)	0.74 (0.53–1.01)		0.82 (0.58–1.18)
Javanese	1.91 (1.52–2.40)			
Urban residence	4.28 (3.22–5.67)	4.51 (3.36–6.06)		1.97 (1.54–2.51)
Married	1.12 (0.88–1.43)	1.13 (0.88–1.44)		1.10 (0.85–1.44)
High school or higher education	7.82 (6.30–9.70)	7.70 (6.19–9.58)	1.58 (1.04–2.41)	4.26 (3.39–5.36)
Monthly household expenditure – higher tertile	2.31 (1.91–2.80)	2.28 (1.88–2.76)	1.94 (1.40–2.69)	1.68 (1.38–2.05)
Physically active	1.54 (1.25–1.91)	1.54 (1.24–1.90)		1.24 (1.00–1.54)
Openness ≥4	0.90 (0.67–1.22)			
Extroversion ≥4	0.87 (0.71–1.05)	0.83 (0.69–1.01)		0.83 (0.68–1.02)
Agreeableness ≥4	1.63 (1.30–2.03)	1.67 (1.33–2.09)		1.61 (1.26–2.05)
Neuroticism ≥4	1.23 (1.02–1.49)	1.24 (1.03–2.09)		1.19 (0.98–1.44)
Enabling				
Have insurance	2.05 (1.69–2.49)	2.01 (1.65–2.44)	1.57 (1.12–2.22)	1.44 (1.18–1.76)
Travel ≥10 min to health service	0.73 (0.55–0.98)	0.70 (0.52–0.94)		0.76 (0.56–1.03)
Participating in social activities	2.12 (1.50–2.98)	2.29 (1.62–3.23)		2.00 (1.38–2.88)
Need				
Postmenopausal	0.76 (0.61–0.96)	0.79 (0.63–0.99)	1.29 (0.90–1.83)	0.58 (0.56–1.03)
Age at menarche ≥14	0.98 (0.93–1.02)	0.98 (0.93–1.03)		0.95 (0.90–1.00)
Co-morbidity score ≥ 2	1.18 (0.92–1.50)	1.09 (0.86–1.40)	1.45 (1.01–2.08)	1.10 (0.85–1.41)
Parent died from cancer	1.50 (0.90–2.50)	1.50 (0.89–2.52)		1.59 (0.99–2.54)
BMI ≥ 25 kg/m^2^	1.15 (0.95–1.39)			1.07 (0.89–1.30)
CES-D ≥ 8	0.68 (0.55–0.85)			

^a^For categorical factors, odds ratios (ORs) were shown for categories displayed in the left-hand column in comparison with the remaining categories as the reference (see Table 2-3)

### Determinants of BSE practice

A number of factors were associated with having performed BSE in the past year in univariable analyses (Table 3). In the multivariable analysis, those associated with higher odds of practicing BSE were living in urban areas (OR: 1.97, 95% CI: 1.54–2.51), had higher education (OR: 4.26, 3.39–5.36), had higher household expenditure (OR: 1.68, 1.38–2.05), had higher agreeable traits (OR: 1.61, 1.26–2.05), had insurance (OR: 1.44, 1.18–1.76), and engaged in social activities (OR: 2.00, 1.38–2.88) (Table 4). A borderline association was shown for physical activity (OR: 1.24, 95% CI: 1.00–1.54) and having menarche at age 14 or older (OR: 0.95, 0.90–1.00 compared to at younger ages) in the multivariable model.

### Sensitivity analyses

Results were also similar when we used BSE at least twice (*N* = 723) instead of once in the past year (*N* = 796) to define women who practiced BSE as the outcome, but this did not alter our findings (data not shown).

## Discussion

Our study identified predisposing, enabling, and need factors associated with awareness of cancer screening and participation in Indonesian women. Most persistent associations were observed for socio-economic determinants, particularly higher education, household expenditure, and ownership of health insurance, which were associated with higher awareness of Pap smears and mammography, and higher odds of participating in Pap smears and BSE. A similar positive association was observed for social activity participation with awareness of Pap smears and BSE practice, whereas distance to nearest health centres was inversely associated with awareness of Pap smears and mammography. Our findings also uncovered associations between personality traits, and Pap-smear awareness and participation and BSE practice which remained when taking into account other determinants.

Despite the increasing cancer burden, most LMICs are yet to publish national guidelines for screening and early detection of breast and cervical cancers [5, 23]. In other LMICs in which national cancer screening programmes have been introduced, such as those in the Middle East and North Africa where screening ranges from 2% to 70% of the at-risk population, improving participation rates remains a challenge [24]. In Sub-Saharan Africa, fewer than 5% of women at risk are estimated to have been screened for cervical cancer [25, 26]. Population-based cervical cancer screening programmes have been in place for more than 10 years in India, however, participation rates are also relatively low [27, 28]. The Indonesian Ministry of Health has recently released new recommendations for preventive measures against cervical and breast cancer (PERMENKES RI No.34/2015) [29]. Approximately 34.5 million Indonesian women are expected to participate in this breast and cervical cancer screening program [29]. According to government recommendations, health promotion should be conducted through public events, media, religious communities, and other civic society channels. Preventive measures include mass screening, mainly for cervical cancer using visual inspection with acetic acid, should be organised as public events. Women in the target age groups may also request examinations for early detection at healthcare facilities. However, no formal invitation for screening is sent to individuals, and there is a lack of clear guidelines regarding the use of mammography. In 2015, only 904,099 (4.94%) women had completed screening and early detection examination for breast and cervical cancer, a similar figure to that observed in this study. The target coverage, however, is 50% by 2019 [29].

Most women in developing countries seek medical care after they develop symptoms. For instance, more than 70% of cervical cancer patients in developing countries visited a hospital once their cancer had already infiltrated the parametrium [30, 31]. A population based-study conducted in Indonesia demonstrated that implementation of small-scale cervical cancer screening project reached only 24% of females in the target group despite the implementation of a mobile screening service to reach more inaccessible areas [32]. We did not find any report evaluating existing programmes or intervention approaches for breast cancer screening. However, it is worth noting that mammography and breast ultrasonography are currently only covered by the national universal health insurance in particular health facilities, which may explain the low cancer screening awareness and participation more generally.

Only a few studies have addressed the role of mental health and personality traits in cancer screening awareness and participation in LMICs [33, 34]. In our study, a higher CES-D score, which is linked to symptoms of depression, was associated with low awareness of Pap smears, but higher odds of BSE practice. This corroborates previous findings linking stress and depression, which are generally more common in individuals of low SES [35], with health-related behaviours [34]. Community support might be required to achieve the desirable level of awareness and participation in cancer screening, especially in women with psychiatric comorbidities. We found associations between higher agreeableness and higher awareness of Pap smears and BSE practice, whereas higher neuroticism was linked with higher awareness of cancer screening. Using a similar approach, two studies also reported associations between personality traits and cancer-related health behaviours, with higher conscientiousness associated with higher participation in bowel and prostate cancer screenings [36, 37]. The positive correlation between conscientiousness and cancer screening awareness did not reach statistical significance in our study. However, our findings support the use of personality-tailored approaches to raise awareness of and participation in cancer screening among women.

As shown in our study and previous ones, sociodemographic determinants including household socio-economic status, ethnicity [38], rural residence, health expenditure, and healthcare access [38] are associated with participation in breast and cervical cancer screening [39]. In addition to these factors, we demonstrated that existing comorbidities were associated with awareness of and participation in screening of breast and cervical cancers. These findings may indicate a complex relationship between health and sociodemographic factors in determining population awareness of, and participation in cancer screening. Therefore, multiple health policies are required to improve the public’s awareness of screening and other initatives as well as the healthcare system’s ability to deliver these initiatives. Interventions may also be needed to advance the skills of primary caregivers for detecting breast and cervical cancer, to promote prompt referrals, to strengthen the system’s capacity for diagnostic imaging, cytology, and histopathology, and to deliver multimodal breast and cervical cancer treatment. Moreover, an effective nationwide cancer registry needs to be established to map cancer incidence and to coordinate screening and evaluation efforts.

The main strength of this study lies in the large number of participants, who live in areas covering 83% of the population in Indonesia in 1993. We were able to account for community clustering and various potential determinants of cancer screening awareness and participation in women. A limitation of this study was that cancer screening awareness only relied on dichotomous responses of questionnaires, without any additional responses allowing for cross-validation and potentially more qualitative work. Additionally, most information was self-reported. However, any misclassification is likely to have been non-differential. We did not use specific cancer questionnaires to measure awareness such as the UK Cancer Awareness Measure [40], since the survey was not originally designed for this particular purpose. Development and validation of a cancer awareness measurement tool that is socioculturally relevant to the Indonesian population is therefore necessary to refine our understanding of the variability in awareness of cancer screening in Indonesia. We were only able to capture mammography use in the past year due to data availability, and this may be a subject of further investigations. It should also be noted that less educated women may have been less familiar with certain medical terminology, although in Indonesia, the terms ‘Pap smear’ and ‘mammography’ are commonly used in the primary care settings [29]. However, we still observed associations between other factors and awareness to either Pap smears or mammography when adjusting for educational levels. Spurious correlations may be of concern when performing multiple comparisons as shown in our study. However, we planned our analyses based on a priori models and our results are explained by potential socioeconomic and health-related mechanisms, and are confirmed by findings from other studies. Therefore, the observed association is unlikely to be spurious [41], although a discrepancy with the strength of the true association is possible due to the small number of participants. Women who responded to screening questionnaires may have different characteristics compared to all women aged 40 and older. Furthermore, although IFLS5 covered most respondents from the original IFLS1 survey, there have been rapid demographic changes in Indonesia [42]. These patterns may reduce the generalisability of our findings. However, demographic transition is well-reflected in the study population, such as the greater number of women living in urban areas in IFLS5 as opposed to the majority living in rural areas in 1993 [42]. Furthermore, this cohort effect is unlikely to affect the internal validity of the results. Finally, our analyses were cross-sectional and only imply associations. Untangling causal associations is necessary to identify key modifiable factors that improve or worsen awareness of and participation in cancer screening.

## Conclusion

We identified a number of factors associated with cervical and breast cancer screening awareness and practice in Indonesia. Improvement of enabling factors such as access to healthcare and social participation may help enhance cancer screening in low-resource settings, particularly among subgroups of women who are socio-economically susceptible to a low awareness of cancer screening. The different associations observed with different personality traits support the potential benefit of employing a range of strategies to promote cancer awareness and participation in Indonesia and potentially other LMICs that lack long-established cancer screening programmes.

## Additional file

Additional file 1: **Table S1.** Demographic characteristics of women based on response to questions on cancer screening. **Figure S1.** Selection of study participants from IFLS5. (PDF 312 kb)
