# Supplementary material for: Determinants of cancer screening awareness and participation among Indonesian women
Source: BMC Cancer. 2018 Mar 6;18:208. doi: 10.1186/s12885-018-4125-z (PMC5838961; doi:10.1186/s12885-018-4125-z)
Supplement: Supplementary file 1 — Table S1. Demographic characteristics of women based on response to questions on cancer screening. Figure S1. Selection of study participants from IFLS5. (PDF 312 kb) [file 12885_2018_4125_MOESM1_ESM.pdf]

Table S1. Demographic characteristics of women based on response to questions on cancer screening

	All women aged ≥40 (N=7,030)	Responded to questions on cancer screening	
		Yes (N=6,320)	No (N=675)
**Age** (years) – mean (SD)	54.50 (11.43)	52.88 (9.95)	69.89 (12.97)
**Ethnicity, Javanese** – N (%)[1]	2893 (41.36)	2863 (45.30)	30 (4.44)
**Urban residence** – N (%)	4045 (57.54)	3684 (58.29)	361 (50.85)
**Married** – N (%)	4828 (68.68)	4594 (72.69)	234 (32.96)
**Education** – N (%)[2]			
Less than high school	4512 (65.06)	3592 (62.66)	570 (86.51)
High school	1923 (27.67)	1864 (29.55)	59 (9.16)
Higher education	506 (7.28)	491 (7.79)	15 (2.33)
**Monthly household expenditure** (million Rp) – mean (SD)	1.03 (0.79)	1.04 (0.78)	0.90 (0.77)

[1]Excluding 10 missing values in the group who did not respond to questions on cancer screening
[2]Excluding 13 missing values in the group who responded and 66 in the group who did not respond to questions on cancer screening

Figure Legends

Figure S1. Selection of study participants from IFLS5.

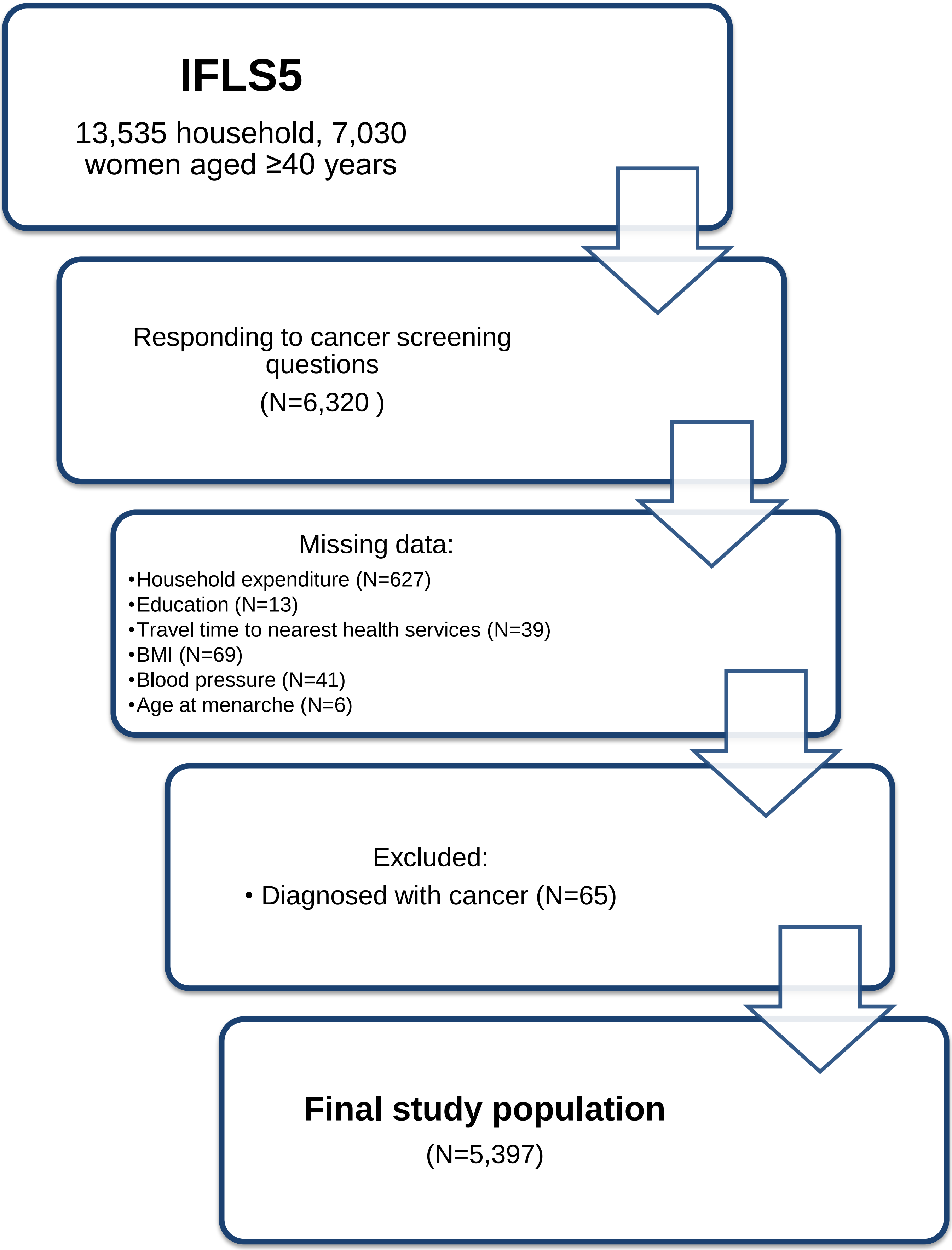
IFLS5
13,535 household, 7,030 women aged ≥40 years
Responding to cancer screening questions
(N=6,320)
Missing data:
•Household expenditure (N=627)
•Education (N=13)
•Travel time to nearest health services (N=39)
•BMI (N=69)
•Blood pressure (N=41)
•Age at menarche (N=6)
Excluded:
• Diagnosed with cancer (N=65)
Final study population
(N=5,397)
